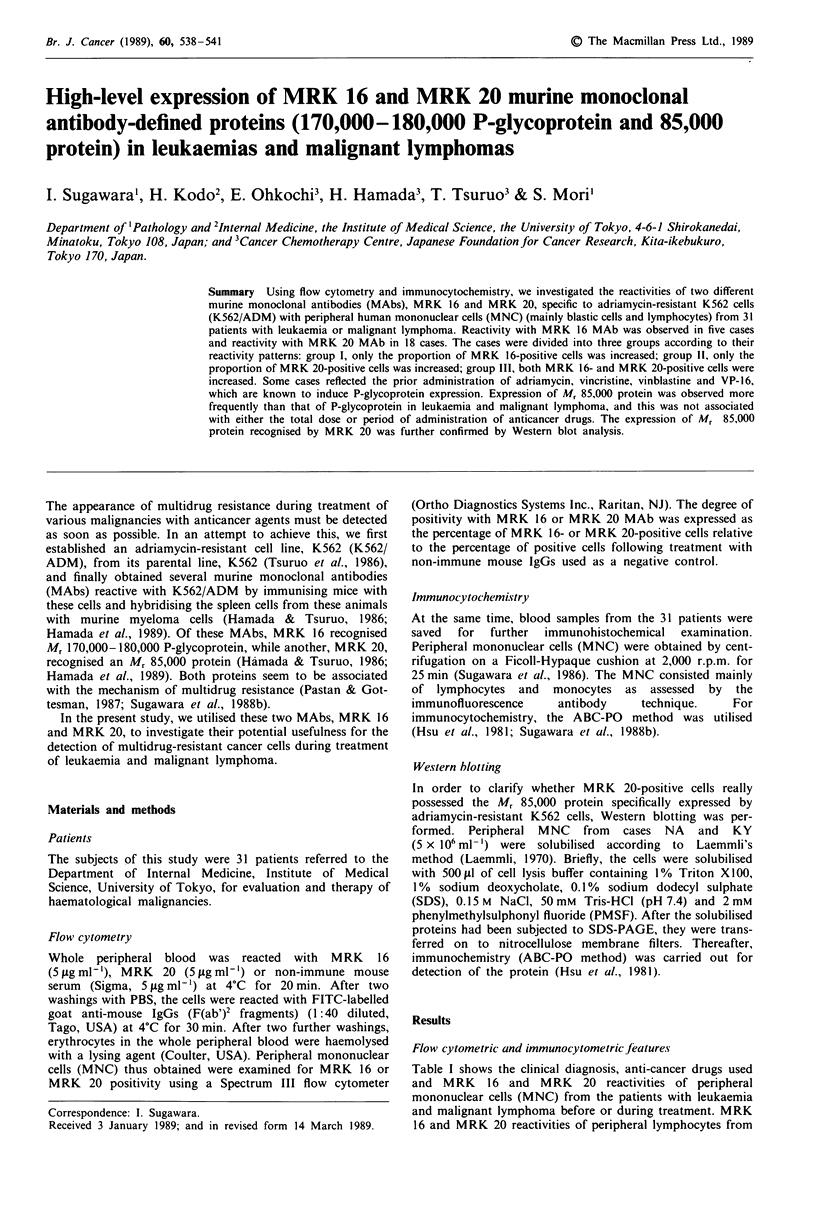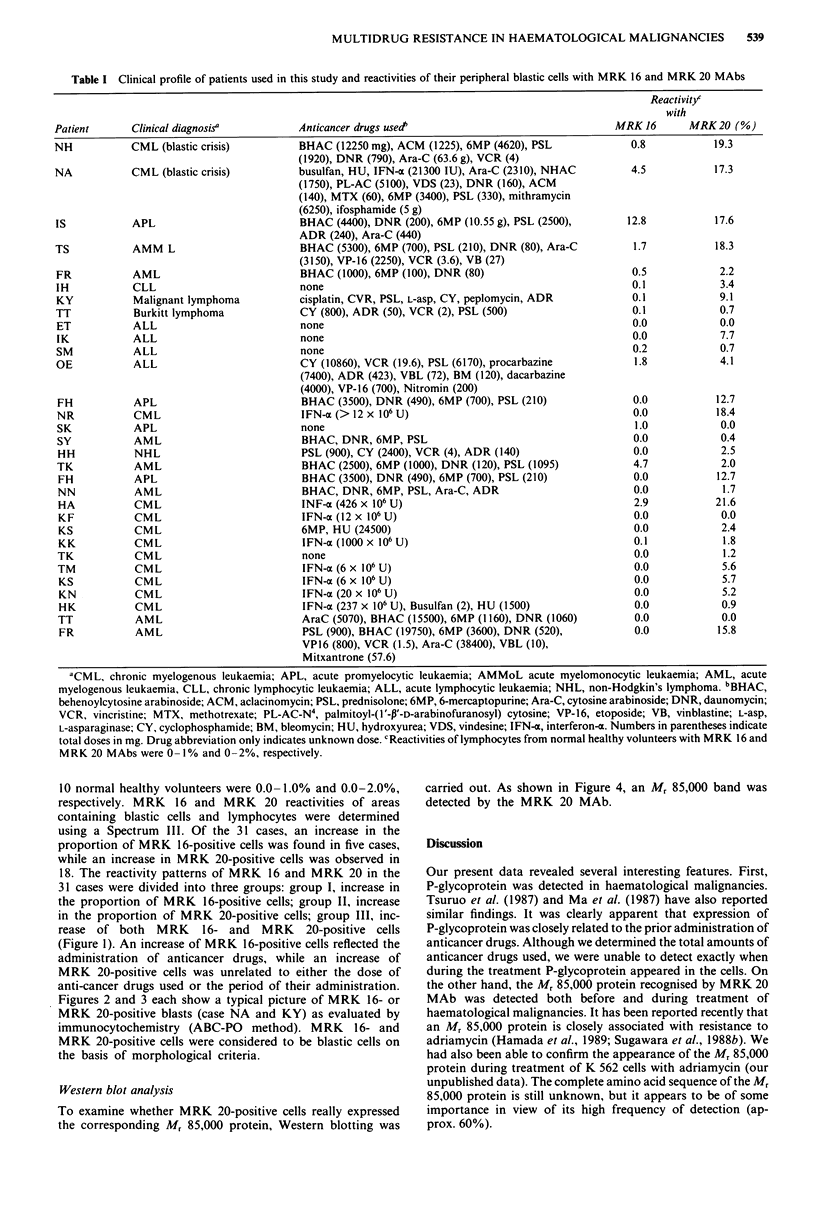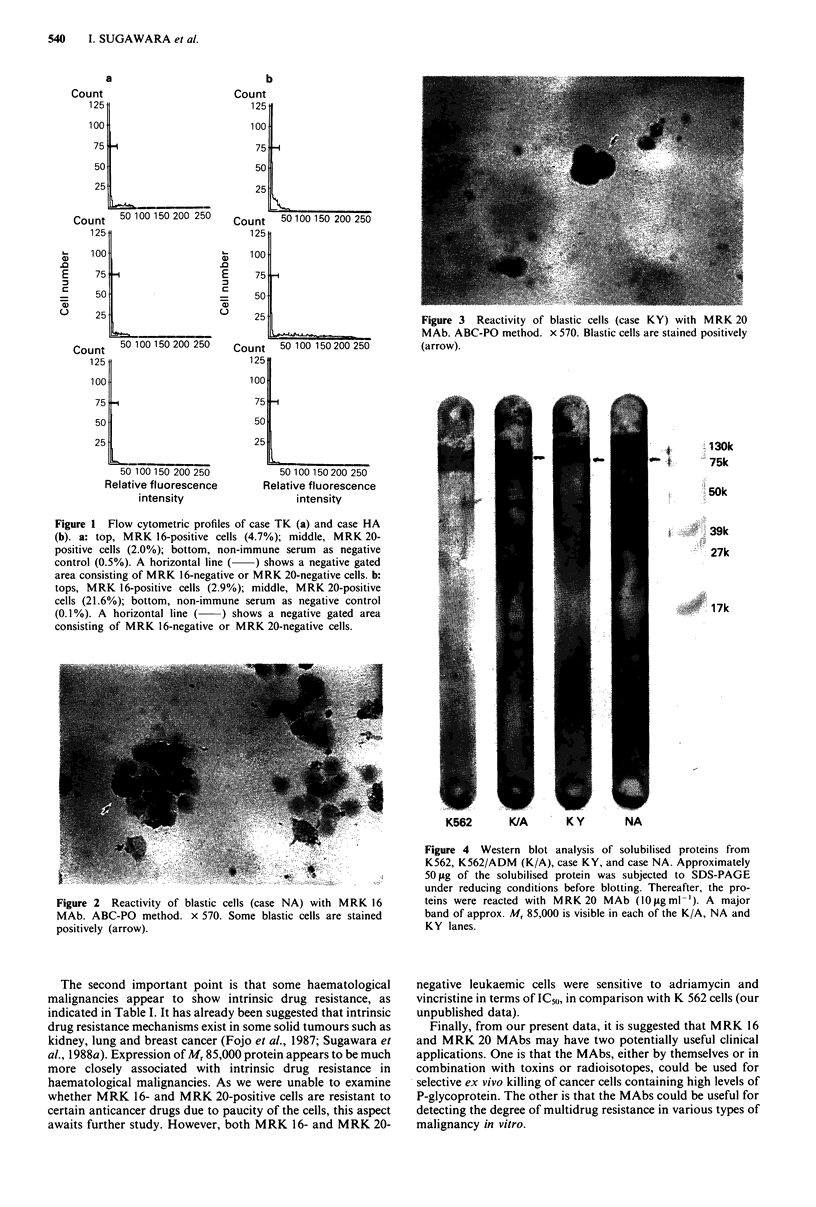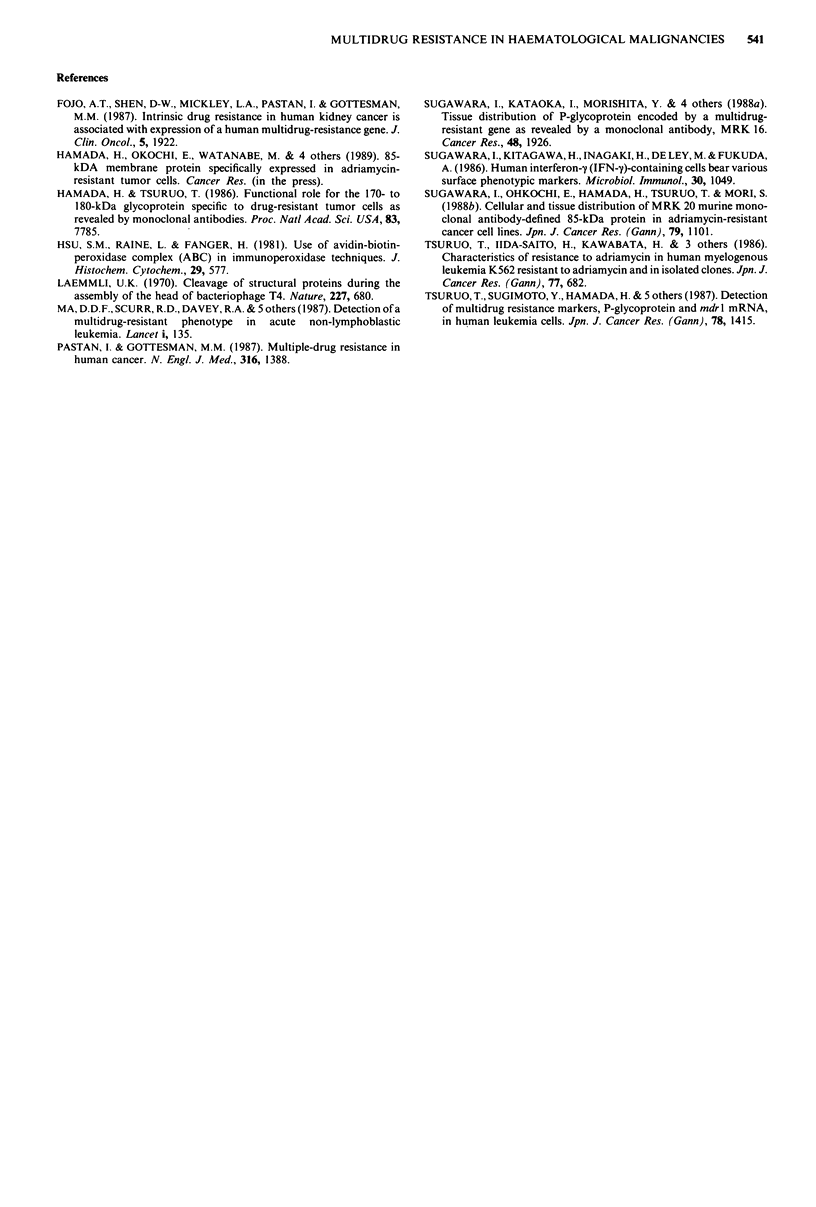# High-level expression of MRK 16 and MRK 20 murine monoclonal antibody-define proteins (170,000-180,000 P-glycoprotein and 85,000 protein) in leukaemias and malignant lymphomas.

**DOI:** 10.1038/bjc.1989.309

**Published:** 1989-10

**Authors:** I. Sugawara, H. Kodo, E. Ohkochi, H. Hamada, T. Tsuruo, S. Mori

**Affiliations:** Department of Pathology, University of Tokyo, Japan.

## Abstract

**Images:**


Br. J. Cancer (1989), 60, 538–541 © The Macmillan Press Ltd., 1989

### High-level expression of MRK 16 and MRK 20 murine monoclonal antibody-defined proteins (170,000–180,000 P-glycoprotein and 85,000 protein) in leukaemias and malignant lymphomas

I. Sugawara[1], H. Kodo[2], E. Ohkochi[3], H. Hamada[3], T. Tsuruo[3] & S. Mori[1]

*Department of [1]Pathology and [2]Internal Medicine, the Institute of Medical Science, the University of Tokyo, 4-6-1 Shirokanedai, Minatoku, Tokyo 108, Japan; and [3]Cancer Chemotherapy Centre, Japanese Foundation for Cancer Research, Kita-ikebukuro, Tokyo 170, Japan.*

**Summary** Using flow cytometry and immunocytochemistry, we investigated the reactivities of two different murine monoclonal antibodies (MAbs), MRK 16 and MRK 20, specific to adriamycin-resistant K562 cells (K562/ADM) with peripheral human mononuclear cells (MNC) (mainly blastic cells and lymphocytes) from 31 patients with leukaemia or malignant lymphoma. Reactivity with MRK 16 MAb was observed in five cases and reactivity with MRK 20 MAb in 18 cases. The cases were divided into three groups according to their reactivity patterns: group I, only the proportion of MRK 16-positive cells was increased; group II, only the proportion of MRK 20-positive cells was increased; group III, both MRK 16- and MRK 20-positive cells were increased. Some cases reflected the prior administration of adriamycin, vincristine, vinblastine and VP-16, which are known to induce P-glycoprotein expression. Expression of $M_r$ 85,000 protein was observed more frequently than that of P-glycoprotein in leukaemia and malignant lymphoma, and this was not associated with either the total dose or period of administration of anticancer drugs. The expression of $M_r$ 85,000 protein recognised by MRK 20 was further confirmed by Western blot analysis.

The appearance of multidrug resistance during treatment of various malignancies with anticancer agents must be detected as soon as possible. In an attempt to achieve this, we first established an adriamycin-resistant cell line, K562 (K562/ADM), from its parental line, K562 (Tsuruo *et al.*, 1986), and finally obtained several murine monoclonal antibodies (MAbs) reactive with K562/ADM by immunising mice with these cells and hybridising the spleen cells from these animals with murine myeloma cells (Hamada & Tsuruo, 1986; Hamada *et al.*, 1989). Of these MAbs, MRK 16 recognised $M_r$ 170,000–180,000 P-glycoprotein, while another, MRK 20, recognised an $M_r$ 85,000 protein (Hamada & Tsuruo, 1986; Hamada *et al.*, 1989). Both proteins seem to be associated with the mechanism of multidrug resistance (Pastan & Gottesman, 1987; Sugawara *et al.*, 1988b).

In the present study, we utilised these two MAbs, MRK 16 and MRK 20, to investigate their potential usefulness for the detection of multidrug-resistant cancer cells during treatment of leukaemia and malignant lymphoma.

### Materials and methods

### Patients

The subjects of this study were 31 patients referred to the Department of Internal Medicine, Institute of Medical Science, University of Tokyo, for evaluation and therapy of haematological malignancies.

### Flow cytometry

Whole peripheral blood was reacted with MRK 16 ($5\,\mu g\,ml^{-1}$), MRK 20 ($5\,\mu g\,ml^{-1}$) or non-immune mouse serum (Sigma, $5\,\mu g\,ml^{-1}$) at 4°C for 20 min. After two washings with PBS, the cells were reacted with FITC-labelled goat anti-mouse IgGs ($F(ab')^2$ fragments) (1:40 diluted, Tago, USA) at 4°C for 30 min. After two further washings, erythrocytes in the whole peripheral blood were haemolysed with a lysing agent (Coulter, USA). Peripheral mononuclear cells (MNC) thus obtained were examined for MRK 16 or MRK 20 positivity using a Spectrum III flow cytometer (Ortho Diagnostics Systems Inc., Raritan, NJ). The degree of positivity with MRK 16 or MRK 20 MAb was expressed as the percentage of MRK 16- or MRK 20-positive cells relative to the percentage of positive cells following treatment with non-immune mouse IgGs used as a negative control.

Correspondence: I. Sugawara.
Received 3 January 1989; and in revised form 14 March 1989.

### Immunocytochemistry

At the same time, blood samples from the 31 patients were saved for further immunohistochemical examination. Peripheral mononuclear cells (MNC) were obtained by centrifugation on a Ficoll-Hypaque cushion at 2,000 r.p.m. for 25 min (Sugawara *et al.*, 1986). The MNC consisted mainly of lymphocytes and monocytes as assessed by the immunofluorescence antibody technique. For immunocytochemistry, the ABC-PO method was utilised (Hsu *et al.*, 1981; Sugawara *et al.*, 1988b).

### Western blotting

In order to clarify whether MRK 20-positive cells really possessed the $M_r$ 85,000 protein specifically expressed by adriamycin-resistant K562 cells, Western blotting was performed. Peripheral MNC from cases NA and KY ($5 \times 10^6\,ml^{-1}$) were solubilised according to Laemmli's method (Laemmli, 1970). Briefly, the cells were solubilised with 500 µl of cell lysis buffer containing 1% Triton X100, 1% sodium deoxycholate, 0.1% sodium dodecyl sulphate (SDS), 0.15 M NaCl, 50 mM Tris-HCl (pH 7.4) and 2 mM phenylmethylsulphonyl fluoride (PMSF). After the solubilised proteins had been subjected to SDS-PAGE, they were transferred on to nitrocellulose membrane filters. Thereafter, immunochemistry (ABC-PO method) was carried out for detection of the protein (Hsu *et al.*, 1981).

### Results

### Flow cytometric and immunocytometric features

Table I shows the clinical diagnosis, anti-cancer drugs used and MRK 16 and MRK 20 reactivities of peripheral mononuclear cells (MNC) from the patients with leukaemia and malignant lymphoma before or during treatment. MRK 16 and MRK 20 reactivities of peripheral lymphocytes from

**Table I** Clinical profile of patients used in this study and reactivities of their peripheral blastic cells with MRK 16 and MRK 20 MAbs

| *Patient* | *Clinical diagnosis*[a] | *Anticancer drugs used*[b] | *Reactivity*[c] *with* *MRK 16* | *MRK 20 (%)* |
|---|---|---|---|---|
| NH | CML (blastic crisis) | BHAC (12250 mg), ACM (1225), 6MP (4620), PSL (1920), DNR (790), Ara-C (63.6 g), VCR (4) | 0.8 | 19.3 |
| NA | CML (blastic crisis) | busulfan, HU, IFN-α (21300 IU), Ara-C (2310), NHAC (1750), PL-AC (5100), VDS (23), DNR (160), ACM (140), MTX (60), 6MP (3400), PSL (330), mithramycin (6250), ifosphamide (5 g) | 4.5 | 17.3 |
| IS | APL | BHAC (4400), DNR (200), 6MP (10.55 g), PSL (2500), ADR (240), Ara-C (440) | 12.8 | 17.6 |
| TS | AMM L | BHAC (5300), 6MP (700), PSL (210), DNR (80), Ara-C (3150), VP-16 (2250), VCR (3.6), VB (27) | 1.7 | 18.3 |
| FR | AML | BHAC (1000), 6MP (100), DNR (80) | 0.5 | 2.2 |
| IH | CLL | none | 0.1 | 3.4 |
| KY | Malignant lymphoma | cisplatin, CVR, PSL, L-asp, CY, peplomycin, ADR | 0.1 | 9.1 |
| TT | Burkitt lymphoma | CY (800), ADR (50), VCR (2), PSL (500) | 0.1 | 0.7 |
| ET | ALL | none | 0.0 | 0.0 |
| IK | ALL | none | 0.0 | 7.7 |
| SM | ALL | none | 0.2 | 0.7 |
| OE | ALL | CY (10860), VCR (19.6), PSL (6170), procarbazine (7400), ADR (423), VBL (72), BM (120), dacarbazine (4000), VP-16 (700), Nitromin (200) | 1.8 | 4.1 |
| FH | APL | BHAC (3500), DNR (490), 6MP (700), PSL (210) | 0.0 | 12.7 |
| NR | CML | IFN-α (> 12 × 10⁶ U) | 0.0 | 18.4 |
| SK | APL | none | 1.0 | 0.0 |
| SY | AML | BHAC, DNR, 6MP, PSL | 0.0 | 0.4 |
| HH | NHL | PSL (900), CY (2400), VCR (4), ADR (140) | 0.0 | 2.5 |
| TK | AML | BHAC (2500), 6MP (1000), DNR (120), PSL (1095) | 4.7 | 2.0 |
| FH | APL | BHAC (3500), DNR (490), 6MP (700), PSL (210) | 0.0 | 12.7 |
| NN | AML | BHAC, DNR, 6MP, PSL, Ara-C, ADR | 0.0 | 1.7 |
| HA | CML | INF-α (426 × 10⁶ U) | 2.9 | 21.6 |
| KF | CML | IFN-α (12 × 10⁶ U) | 0.0 | 0.0 |
| KS | CML | 6MP, HU (24500) | 0.0 | 2.4 |
| KK | CML | IFN-α (1000 × 10⁶ U) | 0.1 | 1.8 |
| TK | CML | none | 0.0 | 1.2 |
| TM | CML | IFN-α (6 × 10⁶ U) | 0.0 | 5.6 |
| KS | CML | IFN-α (6 × 10⁶ U) | 0.0 | 5.7 |
| KN | CML | IFN-α (20 × 10⁶ U) | 0.0 | 5.2 |
| HK | CML | IFN-α (237 × 10⁶ U), Busulfan (2), HU (1500) | 0.0 | 0.9 |
| TT | AML | AraC (5070), BHAC (15500), 6MP (1160), DNR (1060) | 0.0 | 0.0 |
| FR | AML | PSL (900), BHAC (19750), 6MP (3600), DNR (520), VP16 (800), VCR (1.5), Ara-C (38400), VBL (10), Mitxantrone (57.6) | 0.0 | 15.8 |

[a]CML, chronic myelogenous leukaemia; APL, acute promyelocytic leukaemia; AMMoL acute myelomonocytic leukaemia; AML, acute myelogenous leukaemia, CLL, chronic lymphocytic leukaemia; ALL, acute lymphocytic leukaemia; NHL, non-Hodgkin's lymphoma. [b]BHAC, behenoylcytosine arabinoside; ACM, aclacinomycin; PSL, prednisolone; 6MP, 6-mercaptopurine; Ara-C, cytosine arabinoside; DNR, daunomycin; VCR, vincristine; MTX, methotrexate; PL-AC-N[4], palmitoyl-(1′-β′-D-arabinofuranosyl) cytosine; VP-16, etoposide; VB, vinblastine; L-asp, L-asparaginase; CY, cyclophosphamide; BM, bleomycin; HU, hydroxyurea; VDS, vindesine; IFN-α, interferon-α. Numbers in parentheses indicate total doses in mg. Drug abbreviation only indicates unknown dose. [c]Reactivities of lymphocytes from normal healthy volunteers with MRK 16 and MRK 20 MAbs were 0–1% and 0–2%, respectively.

10 normal healthy volunteers were 0.0–1.0% and 0.0–2.0%, respectively. MRK 16 and MRK 20 reactivities of areas containing blastic cells and lymphocytes were determined using a Spectrum III. Of the 31 cases, an increase in the proportion of MRK 16-positive cells was found in five cases, while an increase in MRK 20-positive cells was observed in 18. The reactivity patterns of MRK 16 and MRK 20 in the 31 cases were divided into three groups: group I, increase in the proportion of MRK 16-positive cells; group II, increase in the proportion of MRK 20-positive cells; group III, increase of both MRK 16- and MRK 20-positive cells (Figure 1). An increase of MRK 16-positive cells reflected the administration of anticancer drugs, while an increase of MRK 20-positive cells was unrelated to either the dose of anti-cancer drugs used or the period of their administration. Figures 2 and 3 each show a typical picture of MRK 16- or MRK 20-positive blasts (case NA and KY) as evaluated by immunocytochemistry (ABC-PO method). MRK 16- and MRK 20-positive cells were considered to be blastic cells on the basis of morphological criteria.

*Western blot analysis*

To examine whether MRK 20-positive cells really expressed the corresponding $M_r$ 85,000 protein, Western blotting was carried out. As shown in Figure 4, an $M_r$ 85,000 band was detected by the MRK 20 MAb.

**Discussion**

Our present data revealed several interesting features. First, P-glycoprotein was detected in haematological malignancies. Tsuruo *et al.* (1987) and Ma *et al.* (1987) have also reported similar findings. It was clearly apparent that expression of P-glycoprotein was closely related to the prior administration of anticancer drugs. Although we determined the total amounts of anticancer drugs used, we were unable to detect exactly when during the treatment P-glycoprotein appeared in the cells. On the other hand, the $M_r$ 85,000 protein recognised by MRK 20 MAb was detected both before and during treatment of haematological malignancies. It has been reported recently that an $M_r$ 85,000 protein is closely associated with resistance to adriamycin (Hamada *et al.*, 1989; Sugawara *et al.*, 1988*b*). We had also been able to confirm the appearance of the $M_r$ 85,000 protein during treatment of K 562 cells with adriamycin (our unpublished data). The complete amino acid sequence of the $M_r$ 85,000 protein is still unknown, but it appears to be of some importance in view of its high frequency of detection (approx. 60%).

**Figure 1** Flow cytometric profiles of case TK (**a**) and case HA (**b**). **a:** top, MRK 16-positive cells (4.7%); middle, MRK 20-positive cells (2.0%); bottom, non-immune serum as negative control (0.5%). A horizontal line (——) shows a negative gated area consisting of MRK 16-negative or MRK 20-negative cells. **b:** tops, MRK 16-positive cells (2.9%); middle, MRK 20-positive cells (21.6%); bottom, non-immune serum as negative control (0.1%). A horizontal line (——) shows a negative gated area consisting of MRK 16-negative or MRK 20-negative cells.

**Figure 2** Reactivity of blastic cells (case NA) with MRK 16 MAb. ABC-PO method. × 570. Some blastic cells are stained positively (arrow).

**Figure 3** Reactivity of blastic cells (case KY) with MRK 20 MAb. ABC-PO method. × 570. Blastic cells are stained positively (arrow).

**Figure 4** Western blot analysis of solubilised proteins from K562, K562/ADM (K/A), case KY, and case NA. Approximately 50 μg of the solubilised protein was subjected to SDS-PAGE under reducing conditions before blotting. Thereafter, the proteins were reacted with MRK 20 MAb ($10\,\mu g\,ml^{-1}$). A major band of approx. $M_r$ 85,000 is visible in each of the K/A, NA and KY lanes.

The second important point is that some haematological malignancies appear to show intrinsic drug resistance, as indicated in Table I. It has already been suggested that intrinsic drug resistance mechanisms exist in some solid tumours such as kidney, lung and breast cancer (Fojo *et al.*, 1987; Sugawara *et al.*, 1988*a*). Expression of $M_r$ 85,000 protein appears to be much more closely associated with intrinsic drug resistance in haematological malignancies. As we were unable to examine whether MRK 16- and MRK 20-positive cells are resistant to certain anticancer drugs due to paucity of the cells, this aspect awaits further study. However, both MRK 16- and MRK 20-negative leukaemic cells were sensitive to adriamycin and vincristine in terms of $IC_{50}$, in comparison with K 562 cells (our unpublished data).

Finally, from our present data, it is suggested that MRK 16 and MRK 20 MAbs may have two potentially useful clinical applications. One is that the MAbs, either by themselves or in combination with toxins or radioisotopes, could be used for selective *ex vivo* killing of cancer cells containing high levels of P-glycoprotein. The other is that the MAbs could be useful for detecting the degree of multidrug resistance in various types of malignancy *in vitro*.

### References

FOJO, A.T., SHEN, D-W., MICKLEY, L.A., PASTAN, I. & GOTTESMAN, M.M. (1987). Intrinsic drug resistance in human kidney cancer is associated with expression of a human multidrug-resistance gene. *J. Clin. Oncol.*, **5**, 1922.

HAMADA, H., OKOCHI, E., WATANABE, M. & 4 others (1989). 85-kDA membrane protein specifically expressed in adriamycin-resistant tumor cells. *Cancer Res.* (in the press).

HAMADA, H. & TSURUO, T. (1986). Functional role for the 170- to 180-kDa glycoprotein specific to drug-resistant tumor cells as revealed by monoclonal antibodies. *Proc. Natl Acad. Sci. USA*, **83**, 7785.

HSU, S.M., RAINE, L. & FANGER, H. (1981). Use of avidin-biotin-peroxidase complex (ABC) in immunoperoxidase techniques. *J. Histochem. Cytochem.*, **29**, 577.

LAEMMLI, U.K. (1970). Cleavage of structural proteins during the assembly of the head of bacteriophage T4. *Nature*, **227**, 680.

MA, D.D.F., SCURR, R.D., DAVEY, R.A. & 5 others (1987). Detection of a multidrug-resistant phenotype in acute non-lymphoblastic leukemia. *Lancet* **i**, 135.

PASTAN, I. & GOTTESMAN, M.M. (1987). Multiple-drug resistance in human cancer. *N. Engl. J. Med.*, **316**, 1388.

SUGAWARA, I., KATAOKA, I., MORISHITA, Y. & 4 others (1988*a*). Tissue distribution of P-glycoprotein encoded by a multidrug-resistant gene as revealed by a monoclonal antibody, MRK 16. *Cancer Res.*, **48**, 1926.

SUGAWARA, I., KITAGAWA, H., INAGAKI, H., DE LEY, M. & FUKUDA, A. (1986). Human interferon-γ (IFN-γ)-containing cells bear various surface phenotypic markers. *Microbiol. Immunol.*, **30**, 1049.

SUGAWARA, I., OHKOCHI, E., HAMADA, H., TSURUO, T. & MORI, S. (1988*b*). Cellular and tissue distribution of MRK 20 murine monoclonal antibody-defined 85-kDa protein in adriamycin-resistant cancer cell lines. *Jpn. J. Cancer Res. (Gann)*, **79**, 1101.

TSURUO, T., IIDA-SAITO, H., KAWABATA, H. & 3 others (1986). Characteristics of resistance to adriamycin in human myelogenous leukemia K562 resistant to adriamycin and in isolated clones. *Jpn. J. Cancer Res. (Gann)*, **77**, 682.

TSURUO, T., SUGIMOTO, Y., HAMADA, H. & 5 others (1987). Detection of multidrug resistance markers, P-glycoprotein and *mdr*1 mRNA, in human leukemia cells. *Jpn. J. Cancer Res. (Gann)*, **78**, 1415.